# Striking mycotoxin tolerance and zearalenone elimination capacity of the decaying wood associated yeast *Sugiyamaella novakii* (*Trichomonascaceae*)

**DOI:** 10.1186/s12866-025-04145-7

**Published:** 2025-07-07

**Authors:** Lajos Acs-Szabo, Walter P. Pfliegler, Szilvia Kovács, Cintia Adácsi, Hanna V. Rácz, Enikő Horváth, László A. Papp, Katalin Pappné Murvai, Szabina Király, Ida Miklós, Gábor Péter, Tünde Pusztahelyi, István Pócsi

**Affiliations:** 1 Department of Botany, Faculty of Science and Technology, Institute of Biology and Ecology, University of Debrecen, Egyetem tér 1, Debrecen, H-4032 Hungary; 2 Department of Molecular Biotechnology and Microbiology, Faculty of Science and Technology, Institute of Biotechnology, University of Debrecen, Egyetem tér 1, Debrecen, H-4032 Hungary; 3 Central Laboratory of Agricultural and Food Products, Faculty of Agricultural and Food Sciences and Environmental Management, University of Debrecen, Böszörményi út 138, Debrecen, H-4032 Hungary; 4 Doctoral School of Nutrition and Food Sciences, Faculty of Medicine, University of Debrecen, Egyetem tér 1, Debrecen, H-4032 Hungary; 5 Invasion Biology Division, Health and Safety National Laboratory, HUN- REN Veterinary Medical Research Institute, Hungária krt. 21, Budapest, H-1143 Hungary; 6 Department of Genetics and Applied Microbiology, Faculty of Science and Technology, Institute of Biotechnology, University of Debrecen, Egyetem tér 1, Debrecen, H-4032 Hungary; 7 Doctoral School of Pharmaceutical Sciences, Faculty of Pharmacy, University of Debrecen, Egyetem tér 1, Debrecen, H-4032 Hungary; 8 National Collection of Agricultural and Industrial Microorganisms, Institute of Food Science and Technology, Hungarian University of Agriculture and Life Sciences, Somlói út 14-16, Budapest, H-1118 Hungary

**Keywords:** *Sugiyamaella*, *Trichomonascaceae*, *Fusarium*, Mycotoxin, Yeast, Genomics, Cell wall

## Abstract

**Background:**

Mycotoxin-producing fungal species and their mycotoxins pose a global threat for crop production and for human and animal health. Given the increasing demand for healthier food and feed, alternative non-pesticide approaches for reducing fungal infections in crops and eliminating mycotoxin contamination in feedstock are becoming more prevalent. For such purposes, yeast species can be good candidates. Thus, the present study examined the mycotoxin tolerance and mycotoxin elimination ability of several yeast strains belonging to the *Trichomonascaceae* family.

**Results:**

While none of the tested yeasts inhibited the growth of different *Fusarium* species, several yeast strains exhibited tolerance to *Fusarium* mycotoxins such as deoxynivalenol, zearalenone, T-2 toxin, and fumonisin B1. *Sugiyamaella novakii* strains displayed exceptional tolerance for the tested mycotoxins. Besides, phylogenetic analyses suggested that tolerant species clustered more closely to each other than to the sensitive species. Although whole genome sequencing of *S. novakii* NCAIM Y.00986 revealed several enzyme-coding genes that may have a role in mycotoxin elimination, significant mycotoxin elimination was not achieved in the case of deoxynivalenol, T-2 toxin, and fumonisin B1. However, *S. novakii* successfully eliminated zearalenone, likely due to cell wall adsorption rather than enzymatic degradation.

**Conclusions:**

This study highlights the potential of *S. novakii* for zearalenone detoxification and emphasizes the role of yeast cell walls in mycotoxin mitigation strategies.

**Supplementary Information:**

The online version contains supplementary material available at 10.1186/s12866-025-04145-7.

## Background

Numerous fungal species have the ability to produce mycotoxins, which are harmful secondary metabolites threatening human and animal health [1–3]. Acute or chronic exposure to mycotoxins can result in serious health issues, a worldwide concern according to global trends [4]. The most relevant toxigenic fungal species belong to genera such as *Aspergillus*, *Penicillium*, *Alternaria*, and *Fusarium* [1]. Since the demand for healthier food and feed is constantly growing, alternative (non-pesticide) methods to minimize fungal infections on crops or successfully eliminate mycotoxin contaminations from feedstock are gaining ground [5–7]. Therefore, many studies focus on examining beneficial microbes that are able to hinder fungal contamination and identifying their mechanism, with which they can reduce mycotoxin burden [2, 3, 5–12].

Certain yeast species could be remarkably good candidates for such purposes, since many of them were proved to be potent biocontrol agents (e.g., *Aureobasidium* [13–16], *Debaryomyces* [17–19], *Metschnikowia* [20–22], and *Galactomyces* spp [23])., and several yeasts harbor enzymes, which can degrade or transform mycotoxins into less-toxic or even non-toxic substances [2, 12, 24, 25]. For example, the enzyme trichothecene 3-O-acetyltransferase is involved in self-defense against trichothecenes in certain *Fusarium* species, but functional orthologous genes exist in several yeast species, too [26–28]. That particular enzyme converts trichothecenes into their less toxic 3-acetyl derivatives, which is a common biotransformation of the trichothecene T-2 mycotoxin [27]. Certain fungal and yeast carboxylesterases and aminotransferases are potent against fumonisins (FUMs), while peroxiredoxins, lactonohydrolases, zearalenone hydrolase, and carboxypeptidases could be effective against zearalenone (ZEA) mycotoxin [24, 29–33]. Besides, yeasts can accumulate mycotoxins inside their cells or adsorb mycotoxins with their cell wall, a trait utilized in the decontamination of crop products and feedstock [2, 11, 12].

However, in this regard, there is little information available about the *Trichomonascaceae* family (e.g., *Trichomonascus*,* Blastobotrys*,* Sugiyamaella*,* Spencermartinsiella*, and *Diddensiella*). These yeast species are mainly saprotrophs, but within the *Sugiyamaella* genus, animal symbiotic species can also be found [34–40]. It is known that microbes inhabiting the gastrointestinal tracts of herbivorous animals are often exposed to mycotoxins. Accordingly, the rumen microbiome is able to adapt to utilize toxic secondary metabolites such as mycotoxins [41]. Since one of the most relevant mycotoxin-producing genera is *Fusarium* [42] and the *Trichomonascaceae* yeasts inhabit niches common with them [43], adaptation in these species to higher mycotoxin burden may be plausible. Furthermore, there is already evidence suggesting potent mycotoxin-modifying ability of certain *Trichomonascaceae* yeasts [27]. Unfortunately, most of the publications on those yeast species are taxonomic descriptions, and in connection with mycotoxins, there cannot be found many. Therefore, the examination of lesser-known groups of yeasts might uncover new species or strains that can tolerate and eliminate mycotoxins.

Accordingly, the aim of the present study was to compare some *Trichomonascaceae* yeast species regarding antimicrobial activity against *Fusarium* species, mycotoxin tolerance, and elimination capacity against *Fusarium* mycotoxins like deoxynivalenol (DON), ZEA, T-2, and fumonisin B1 (FB1). If substantial mycotoxin tolerance or mycotoxin elimination capacity can be observed in the case of some yeast strains during the laboratory experiments, the analyses of their genome sequence may help to understand the underlying phenomena. For instance, if a tested yeast strain has a set of enzymes that are capable of biodegrading the given mycotoxin according to international literature, then there is a chance that the observed decrease in the mycotoxin levels may be a result of enzymatic processes. However, if the activity of enzymes could be excluded, it is possible that mycotoxin reduction was achieved by cell-wall adsorption. Understanding these phenomena requires the application of a multidisciplinary approach combining microbial, biochemical, and bioinformatics methods.

## Materials and methods

### Strains and media

In this study, 29 strains of 10 yeast species belonging to the *Trichomonascaceae* family were obtained from the Hungarian National Collection of Agricultural and Industrial Microorganisms (NCAIM: https://ncaim.etk.szie.hu/) culture collection (Table 1). All the included yeast strains were isolated in Hungary. *Fusarium* species used in this study are also listed in Table 1. To maintain genetic stability, all the strains were stored as cryopreserved at −80℃ and occasionally resurrected for the experiments. Malt Extract Glucose Agar (MXGA: 3% malt extract, 2% D-glucose, 2% agar; VWR chemicals) and Yeast Peptone Dextrose Agar (YPDA: 1% yeast extract, 2% peptone, 2% D-glucose, and 2% agar; VWR chemicals) were applied to short-term maintenance of yeasts and standard microbial tests. Potato Dextrose Agar (PDA: 0,4% potato infusion powder, 2% D-glucose, 2% agar; VWR chemicals) was used for the short-term maintenance of *Fusarium* strains and induction of sporulation [42]. YPD (YPDA without agar), MXGB (MXGA without agar) culture broth, and Phosphate Buffered Saline (VWR^®^ Biotechnology Grade Phosphate Buffered Saline (PBS) Tablets, pH: 7.4) solution were applied to mycotoxin tolerance and elimination studies [44].

**Table 1:** Yeast and fungal strains used in the study

Species	Identifier*
*Trichomonascus apis*	NCAIM Y.01848
*Trichomonascus apis*	NCAIM Y.01849
*Trichomonascus apis*	NCAIM Y.01850
*Trichomonascus apis*	NCAIM Y.01851
*Blastobotrys aristatus*	NCAIM Y.02021
*Blastobotrys indianaensis*	NCAIM Y.02066
*Sugiyamaella chiloensis*	NCAIM Y.01586
*Sugiyamaella chiloensis*	NCAIM Y.02127
*Sugiyamaella japonica*	NCAIM Y.02133
*Sugiyamaella novakii*	NCAIM Y.00986
*Sugiyamaella novakii*	NCAIM Y.00987
*Sugiyamaella paludigena*	NCAIM Y.02116
*Spencermartinsiella europaea*	NCAIM Y.01815
*Spencermartinsiella europaea*	NCAIM Y.01816
*Spencermartinsiella europaea*	NCAIM Y.01817
*Spencermartinsiella europaea*	NCAIM Y.01818
*Spencermartinsiella europaea*	NCAIM Y.01819
*Spencermartinsiella europaea*	NCAIM Y.01897
*Spencermartinsiella europaea*	NCAIM Y.01961
*Spencermartinsiella europaea*	NCAIM Y.01962
*Spencermartinsiella europaea*	NCAIM Y.01963
*Spencermartinsiella europaea*	NCAIM Y.01964
*Spencermartinsiella ligniputridi*	NCAIM Y.01936
*Spencermartinsiella ligniputridi*	NCAIM Y.01991
*Spencermartinsiella ligniputridi*	NCAIM Y.01992
*Spencermartinsiella ligniputridi*	NCAIM Y.01993
*Diddensiella caesifluorescens*	NCAIM Y.01947
*Diddensiella caesifluorescens*	NCAIM Y.01948
*Diddensiella caesifluorescens*	NCAIM Y.01949
*Fusarium graminearum*	FGSC 9075
*Fusarium oxysporum f. sp. lycopersici*	FGSC 9935
*Fusarium verticillioides*	FGSC 7600
*Fusarium proliferatum*	NRRL 62905

*NCAIM National Collection of Agricultural and Industrial Microorganisms, Budapest, Hungary; *NRRL* National Center for Agricultural Utilization Research in Peoria, Illinois, *FGSC* Fungal Genetics Stock Center, Manhattan, USA

### Antagonism to fusarium species

Co-cultivation tests for microbial antagonism of yeast strains against *Fusarium graminearum*,* Fusarium oxysporum*,* Fusarium verticilloides*, and *Fusarium proliferatum* (Table 1) were performed. Briefly, the *Fusarium* strains were cultivated on PDA for 7 to 11 days at 25℃. Thereafter, fungal conidia were collected in sterile water, and spore suspensions (1 × 10^4^/ml) were inoculated into MXGA-containing single-well plates. In parallel, 1 × 10^7^/ml yeast cell suspensions were made in sterile water from overnight cultures incubated on YPDA medium at 25℃. 10–10 µl yeast suspensions were spotted onto the dried spore-suspension containing MXGA plates and incubated at 25℃ and 30℃. According to our preliminary cultivation tests, the MXGA was the most suitable common medium that promotes the growth of both the yeasts and *Fusarium* species. Microbial antagonism was assessed by the presence or absence of clear zones around the yeast colonies where the *Fusarium* species could not grow [21, 45, 46]. Contact inhibition, when a yeast strain forms a dense colony or biofilm that halts the growth of the *Fusarium* species, was not considered [46]. Microbial antagonisms were checked daily for at least 10 days.

### Mycotoxin tolerance studies

From overnight cultures, 10 µl of the yeast suspensions were added into 190 µl MXGB broth in a microtiter plate, and different concentrations (up to 2 ppm) of DON, ZEA, T-2, and FB1 (BIOPURE, Romer Labs, Tulln, Austria) solutions were applied. The yeast strains were incubated at 25℃ in a microtiter plate reader (Synergy HTX Multimode Reader, BioTek, Winooski, VT, USA) for 24 h, and the optical density was read hourly at 570 nm after intense shaking (30 s). The growth curves of the untreated cultures (without mycotoxins) were compared to the mycotoxin-treated cultures, considering the former values 100%. The tests were repeated three times.

### Mycotoxin detection with HPLC

HPLC detection of DON and ZEA mycotoxins was performed with a Dionex Ultimate 3000 (Thermo Scientific) equipment or with a Hitachi Elite LaChrom HPLC (San Jose, CA, USA) equipment [47]. For DON measurement, the filtrated supernatant samples were loaded onto a Phenomenex (Torrance, CA, USA) RP-C18 column (125 × 4 mm, 5 μm) and detected with a diode array detector in UV 218 nm with acetonitrile: water (10:90) eluent. For ZEA detection, Phenomenex RP C18 column (150 × 4.5 mm, 5 μm) was used, with ex360 nm, em440 nm fluorescence detector, and acetonitrile: water: methanol (46: 46: 8) elution. The performance, limit of detection, linear range, and reproducibility of the applied HPLC methods were determined by spiking of mycotoxins of different concentrations (*n* = 8). For DON, LOD was 50 µg l^−1^. For ZEA, LOD was 2 µg l^−1^. A linear range of up to 50 mg kg^−1^ was detected. The relative standard deviation (RSD) was calculated and it was found to be below 10% in all cases.

### Mycotoxin detection in ELISA assay

The detection of FB1 and T-2 mycotoxins was performed using AgraQuant^®^ Fumonisin ELISA (LOD: 0.2 mg kg^−1^, LOQ: 0.025 mg kg^−1^, quantitation range: 0.2-5 mg kg^−1^) and AgraQuant^®^ T-2 Toxin ELISA tests (LOD: 0.01 mg kg^−1^, LOQ: 0.020 mg kg^−1^, quantitation range: 0.02–0.5 mg kg^−1^), respectively, according to the manufacturer’s instructions (Romer Labs, Tulln, Austria). Subsequently, using the BioTek Synergy HTX Multimode Reader (BioTek, Winooski, VT, USA), the samples were measured at 450 nm (*n* = 3, RSD < 5%).

### Mycotoxin elimination studies

All 2 ppm mycotoxin-supplemented yeast samples (1 × 10^9^ cells) were incubated in 1 ml MXGB, YPD or PBS for 1 to 6 h at 25℃ with shaking (250 rpm), centrifuged (8000 rpm, 10 min, 4℃) (Sigma 3K30, Germany), and the supernatants were removed. The supernatants were treated with methanol in a 1:1 ratio, vortexed at high speed, and filtered through Spartan 0.45 μm filter discs (Millipore, Merck, Darmstadt, Germany). The filtrates were analyzed with HPLC or with the ELISA method as described earlier. For all measurements, BIOPURE mycotoxin standard solutions (Romer Labs, Tulln, Austria) were used in appropriate dilution. All assays were performed in triplicate, and positive controls (without cells) and negative controls (without mycotoxin) were included. Mycotoxin elimination was evaluated by comparing the changes in the mycotoxin concentrations measured in the case of the mycotoxin-supplemented yeast samples with the mycotoxin concentrations of the positive controls. Slight modifications of the mycotoxins, which cannot be efficiently detected by standard HPLC, were out of the scope of this study. If the mycotoxin concentration was not changed after the yeast treatment, we considered no mycotoxin elimination to occur.

If mycotoxin elimination can be observed in the presence of viable yeast cells, heat-inactivated cells and cell debris were also tested for mycotoxin elimination. Heat inactivation was performed by incubating the viable yeast cells at 100℃ for 15 min (Thermo Shaker, Biosan TS-100) in 1 ml sterile water to inactivate the yeast’s enzymes. Cell debris was produced by disrupting the yeast cells with glass beads with a benchtop disruptor (Disruptor Genie, Scientific Industries Inc., USA). Right after that both sample types were centrifuged (13,000 rpm, 5 min, 25℃) (Heraeus Biofuge Pico, Kendro, Germany), and the pellets were washed with 1 ml PBS three times. Finally, the pellets were resuspended in 1 ml PBS.

### Oxford nanopore sequencing and genome assembly

For Oxford Nanopore sequencing, genomic high molecular weight DNA was isolated with the Quick-DNA™ HMW MagBead Kit (Zymo Research, Irvine, CA, USA) according to the company’s instructions. Cell lysis was performed with R-Zymolyase (Zymo Research; 50 units of Zymolyase and 5 units of RNase A in 500 µl end volume, ~10^8^ cells treated at 37 °C for 60 min). Library construction was performed with the Ligation Sequencing Kit (SQK LSK109; Oxford Nanopore, Oxford, UK). The library was sequenced on a MinION R9.4.1 Flow Cell with approximately 2.6 Gb of data before filtering. Super accurate base-calling of the resulting FAST5 sequencing files was performed with Guppy v6.4 software provided by the company. The genome was assembled by using the FASTQ files after base-calling with the LRSDAY v1.6 pipeline [48] combined with other software. After adapter trimming and filtering, Flye v2.9 was used for assembly [49]. Long-read polishing was performed with Racon v1.4 and Medaka v1.4 [50]. The LRSDAY pipeline was also used to annotate the genome (using MAKER [51]). The assembly was deposited to GenBank, accession number JBJGDZ000000000, annotation was deposited in FigShare (doi: 10.6084/m9.figshare.27640185). The raw sequencing reads are deposited under NCBI BioProject PRJNA1183396. BUSCO version 5.1.2 was used to check the completeness of the assembly using 76 related yeast genomes for comparison.

### Whole genome alignments

Pairwise and multiple whole genome alignments were performed with the Mauve aligner (version 2015-02-26) using the progressiveMauve algorithm with the option “use seed families”, otherwise standard parameters were used [52]. YASS was also used for pairwise genome alignments with the following parameters: E value: 1.0 × 10^−30^; X-drop: 50; window range: 100–200,000; window incr.: 2×; hit criterion: double and default parameters were used for the others [53]. In the whole genome conservation estimated with YASS, only alignments larger or equal to 1000 nucleotides were considered [54].

### Phylogenetic tree construction

Mycobank database (https://www.mycobank.org/Simple%20names%20search) (accessed on 23.04.2024) search was applied to investigate the *Sugiyamaella* genus. The 26S rDNA sequence of the *Sugiyamaella lignohabitans* NRRL YB-1473 reference strain was obtained from the NCBI Gene database (https://www.ncbi.nlm.nih.gov/gene/). This particular DNA sequence (NCBI accession: NG_042428.1) was used as a query for BLASTn searches to find 26S rDNA sequences of other type strains [55] of *Sugiyamaella* species in the RefSeq database [56]. For phylogenetic tree creations, manually adjusted workflows were used on the website of Phylogeny.fr (https://www.phylogeny.fr/alacarte.cgi) [57]. In brief, the obtained sequences were aligned with MUSCLE [58], and the ambiguous regions were removed with GBLOCKS [59]. Well-aligned sites of the multiple alignments were used for maximum-likelihood (ML) phylogenetic analysis performed with PhyML v3.0 [60] and for neighbor-joining (NJ) with BioNJ [61]. For the PhyML analysis, the GTR substitution model was chosen, and the number of substitution rate categories was adjusted to 4. The proportion of invariable sites and gamma-shape parameters were both estimated. aLRT was used for estimating branch support [62]. In the case of BioNJ, the K2P substitution model was applied, and 100 bootstrap replicates were created for branch support. The created phylogenetic trees were displayed and edited with FigTree v1.4.2 (http://tree.bio.ed.ac.uk/software/figtree/).

### Identifying potential mycotoxin-degrading enzymes

A literature survey was performed to find potential mycotoxin-degrading enzymes, which proved to be effective against *Fusarium* mycotoxins. Then, the proteome of *S. lignohabitans* (5132 proteins) was searched for such putative enzymes in the Uniprot database (https://www.uniprot.org/uniprotkb?query=sugiyamaella). The protein sequences of the identified enzymes were downloaded and used as queries for tBLASTn searches in the raw genomic sequence of the strain *S. novakii*. Thereafter, the found sequences or sequence fragments were compared to the previously created gene annotations (performed with MAKER) and to the predicted ORFs in SnapGene Viewer (https://www.snapgene.com/snapgene-viewer).

## Results

### Microbial inhibition and mycotoxin tolerance studies

In this study, we have tested 29 strains of 10 yeast species for microbial antagonism against four *Fusarium* species (Table 1). Considering our criteria for microbial antagonism (see methods), none of the yeast strains were able to successfully inhibit the growth of the *Fusarium* species in the tested laboratory conditions (Supplementary Table S1). Nevertheless, microbial antagonism and mycotoxin tolerance depend on different molecular mechanisms; therefore, despite the failed inhibition ability, the yeast strains may show good tolerance and elimination capability for *Fusarium* mycotoxins.

**Fig. 1:**
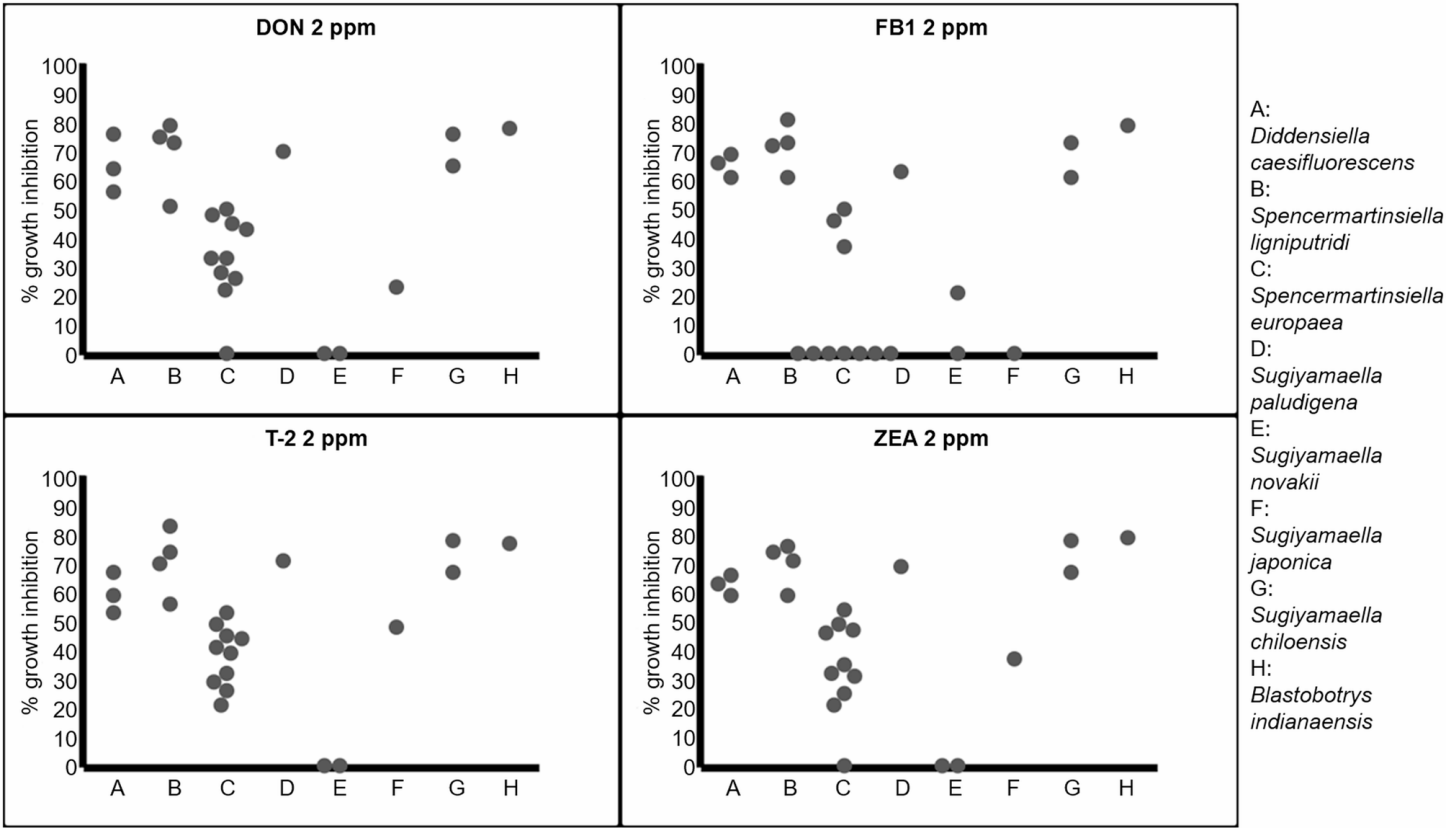
Mycotoxin tolerance of *Trichomonascaceae* yeast strains. While most of the strains showed substantial sensitivity to the tested mycotoxins, one strain of *S. eropaea* and the two *S. novakii* strains exhibited striking tolerance for *Fusarium* mycotoxins. The yeast strains were incubated for 24 hours at 25°C in MXGB broth with or without mycotoxin supplementation. The growth curve was determined at 570 nm for 24 h. Growth changes were evaluated compared to the untreated yeast cultures. The tests were repeated three times

Accordingly, the yeast strains were tested for tolerance of *Fusarium* mycotoxins like DON, ZEA, T-2, and FB1. Since the *Trichomonascus apis* and *Blastobotrys aristatus* strains showed a highly flocculent morphology and did not produce homogeneous cell biomass in liquid conditions in MXGB, those strains were excluded from the following mycotoxin tolerance studies. Some of the tested strains performed better under mycotoxin stress than others, but most of them suffered high, up to 83%, growth inhibition compared to the untreated controls under 24 h culturing time (Fig. 1 and Supplementary Table S2). The mean inhibition values were 46% for DON, 47% for ZEA, 49% for T-2, and 38% for FB1. From the tested 24 yeast strains, only certain strains of three species were able to show substantial tolerances (inhibition less than 50%) to 2 ppm DON, ZEA, T-2, and FB1. While *Sugiyamaella japonica* and most of the *Spencermartinsiella europaea* strains showed moderate sensitivity, one strain of *S. europaea* and the two *Sugiyamaella novakii* strains proved to be highly tolerant to the different *Fusarium* mycotoxins. That peculiar *S. eropaea* strain (NCAIM Y.01961) showed a 21% growth inhibition to T-2, and the ex-type strain (NCAIM Y.00986) of *S. novakii* suffered a 21% growth inhibition in the presence of FB1; otherwise, neither of the *S. novakii* strains nor the *S. eropaea* strain (NCAIM Y.01961) showed any inhibition at all for other mycotoxins. The other strains belonging to the *Trichomonascaceae* family exhibited substantial sensitivity (more than 60% inhibition) to the tested mycotoxins (Fig. 1 and Supplementary Table S2).

We observed that the four tested *Sugiyamaella* species performed quite differently in the mycotoxin tolerance studies. While *S. japonica* and *S. novakii* performed moderately and excellently well, respectively, *S. chiloensis* and *S. paludigena* showed high sensitivity to *Fusarium* mycotoxins (Fig. 1 and Supplementary Table S2). To test if the species that exhibited similar behavior (regarding mycotoxin tolerance) were more closely related or not, we have performed phylogenetic analyses.

### Phylogenetic analyses of the *Sugiyamaella* genus

As stated in the Mycobank database, the genus *Sugiyamaella* contains 30 species with legitimate nomenclature (https://www.mycobank.org/Simple%20names%20search). Since only one *Sugiyamaella* species (*S. lignohabitans*) is available with a sequenced and annotated genome, that species was used as a reference for sequence searches. Accordingly, the partial sequence of the 26S rDNA of *S. lignohabitans* was used as a query for BLASTn searches at the NCBI RefSeq database [56]. The BLASTn search identified 13 *Sugiyamaella* species with high overall scores and excellent alignment coverage values. According to the BLASTn results, *S. novakii* was the second most similar to *S. lignohabitans* (pairwise identity 99.08%) beside *S. marionensis* (pairwise identity 99.51%). Although *S. chiloensis* and the type species of the *Sugiyamaella* genus, *S. smithiae* [63] did not belong to the 14 most similar *Sugiyamaella* species according to BLASTn results, they were also included in the phylogenetic analyses.

**Fig. 2:**
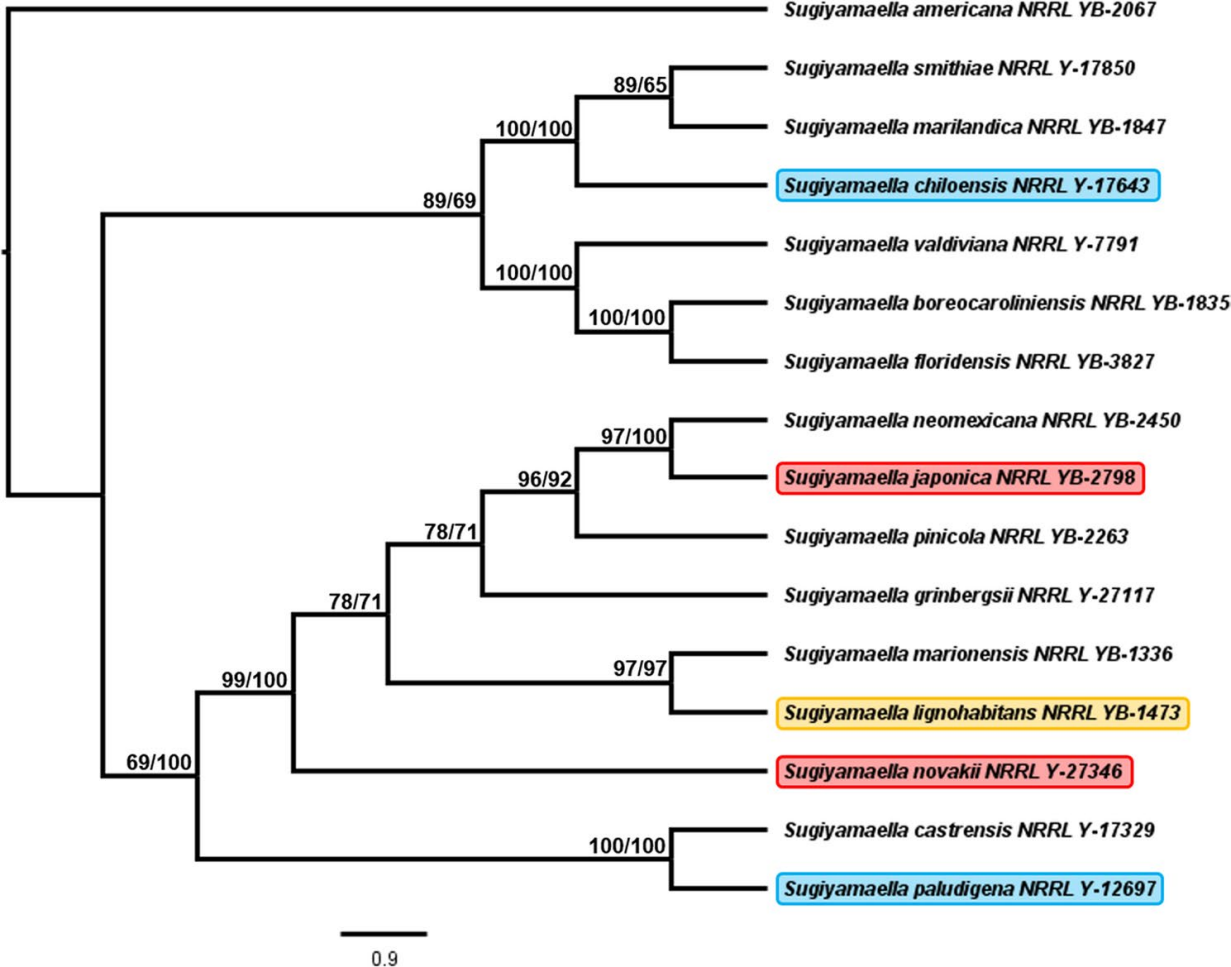
Cladogram of selected species from the genus *Sugiyamaella*. The species used in this study are highlighted in colorful rectangles. Red highlights indicate mycotoxin-tolerant species; blue highlights depict mycotoxin-sensitive species. *S. lignohabitans* (orange highlight) was used for data mining. The mycotoxin-tolerant species are more closely related to each other than to the sensitive species. Since the two methods (ML and NJ) that were used for tree building resulted in identical topology but slightly different branch lengths, the results were depicted as a single cladogram tree. Although the branch length on a cladogram tree is not informative, the original phylograms strengthen the above-mentioned assumption regarding relatedness (data not shown). The trees were created with PhyML 3.0 [60] and BioNJ [61] based on the 26S rDNA sequences of the ex-type strains. Branch supports were estimated with aLRT (ML) and 100 bootstrap replicates (NJ)

The created phylogenetic trees were based on 3123 well-aligned nucleotide sites from the selected sequences, and the two different approaches (ML and NJ) resulted in identical results (Fig. 2). Interestingly, the two mycotoxin-tolerant species (*S. japonica* and *S. novakii*) located more closely to each other than to the sensitive species (*S. chiloensis* and *S. paludigena*) (Fig. 2). Since *S. lignohabitans* clustered with the mycotoxin-tolerant species on the same branch, it allowed us to perform preliminary genome mining to identify potential mycotoxin-degrading enzymes using the annotated sequence of *S. lignohabitans* [64].

### Identification of potential mycotoxin eliminating enzymes in *S. lignohabitans*

Based on the scientific literature, a handful of enzymes produced by bacteria or fungi have been identified that have the ability to transform or degrade *Fusarium* mycotoxins [2, 8, 10, 12, 24, 25, 28–33, 41, 65–70].

Accordingly, 30 enzymes were identified in the proteome of *S. lignohabitans* at the Uniprot database either by the annotated molecular function or by BLASTp searches (Supplementary Table S3). A putative orthologue of AYT1 was identified, which may have a role in transforming the T-2 mycotoxin. For DON elimination, 10 individual aldo-keto reductases were found, and 4 carboxylesterases/carboxylic ester hydrolases and 2 aminotransferases were found for potential FB1 elimination. Although the zearalenone hydrolase (ZDH101) orthologue was not found in the genome of *S. lignohabitans*, several carboxypeptidases (8), peroxidases (4) and a glucosyltransferase (1) were identified, which could have a potential role in ZEA elimination.

Since *S. lignohabitans* might be one of the closest relatives of *S. novakii*, the latter species could also harbor several enzymes that may have roles in the excellent tolerance of the yeast strains for *Fusarium* mycotoxins.

###  Whole genome sequencing and chromosome-level assembly of the *S.novakii* NCAIM Y.00986

For the identification of potential mycotoxin-eliminating enzymes in the genome of *S. novakii*, we have sequenced the ex-type strain (NCAIM Y.00986) of the species.

High molecular weight genomic DNA was isolated from the yeast strain, and whole genome sequencing was performed with the Oxford Nanopore system (Table 2). The assembly comprised five contigs with an overall length of 15,376,747 bases after trimming and polishing, in which four contigs ranged from 2,490,460 to 5,328,050 bases (chromosomes) and a small contig that comprises 29,111 bases (mitochondrial DNA). Across the four chromosomes, 4799 coding genes and 165 tRNA sequences were identified with MAKER. BUSCO assembly completeness was found to be 94.0%.

**Table 2:** Comparison of the two different assemblies of the *S. novakii* strain NCAIM Y.00986

Reference	This study	Opulente et al. [71]
Assembly method	Flye v2.9, Racon v1.4 and Medaka v1.4 polishing	DISCOVAR v. r51885
Genome coverage	~ 169×	~ 67.5×
Sequencing technology	Oxford Nanopore	Illumina HiSeq
Total sequence length	15,376,747	15,264,554
Number of scaffolds	0	238
Scaffold N50	0	208,095
Scaffold L50	0	23
Number of contigs	5	243
Contig N50	-	188,531
Contig L50	-	26
Total number of chromosomes	4 (2.490460 Mb; 2.620666 Mb; 4.908360 Mb; 5.328050 Mb) + mitochondrium (29.211 Kb)	-
SNPs*	280	
Number of Gaps*	484	815
Total bases extra in assembly*	230,886	8,530

*Comparisons were done with the Mauve aligner [52]

**Fig. 3:**
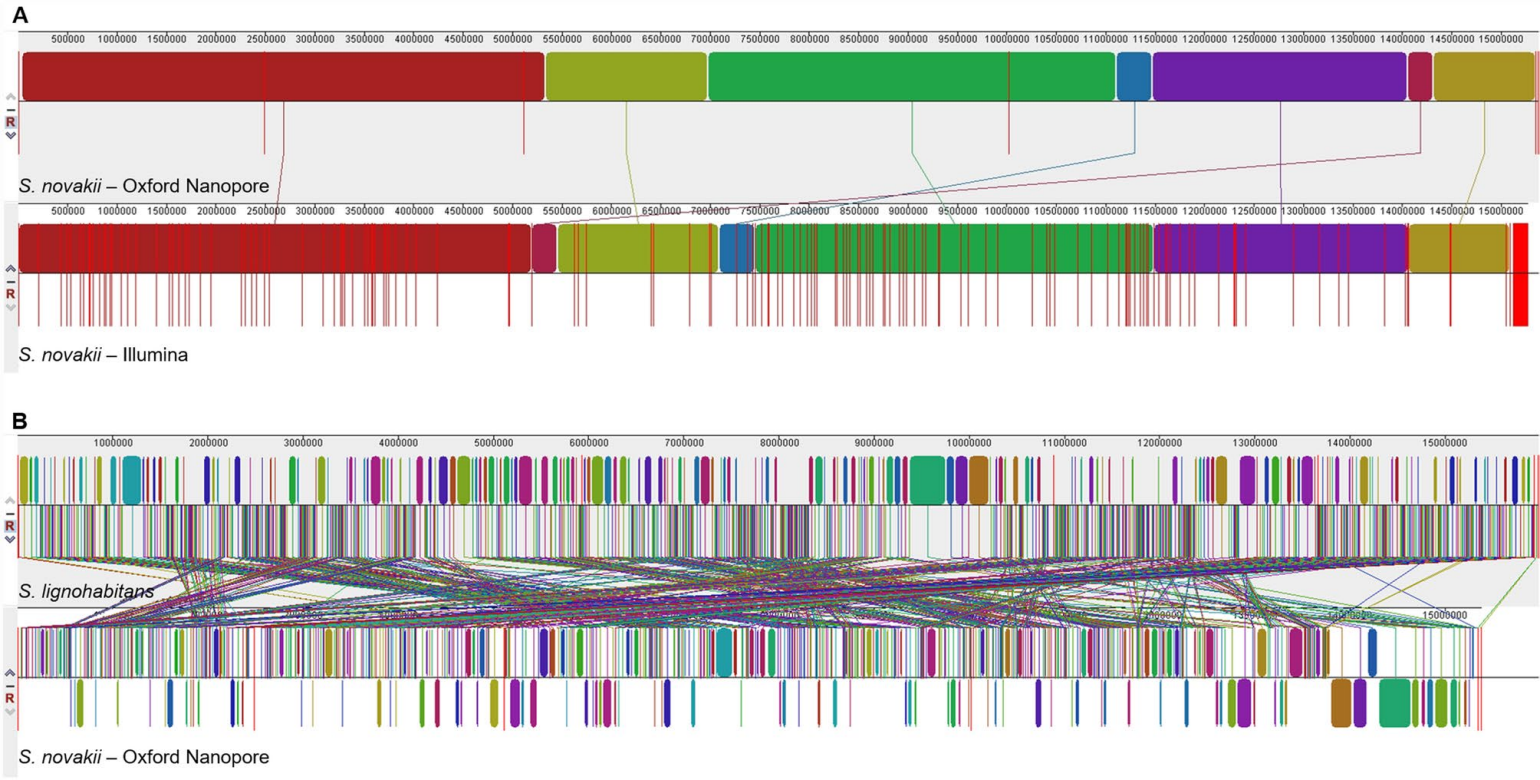
Whole genome alignments performed with the Mauve aligner [52]. (**A**) Whole genome comparison of the different assemblies of the *S. novakii* strain NRRL Y-27346 (NCAIM Y.00986). Alignment and reordering of the contigs of the GCA_030575075.1 Illumina assembly (lower lane) was done with the Mauve aligner using our Oxford Nanopore assembly (upper lane). Since the two assemblies were substantially similar, we considered our genome assembly as valid. Colorful rectangles represent locally collinear blocks (LCBs). Red vertical lines indicate contig boundaries. (**B**) Whole genome alignment of *S. lignohabitans* (upper lane) and *S. novakii* (lower lane). Although the Mauve alignment indicated that several rearrangement events could have occurred on the chromosomes of the species, the overall genome conservation seems to be high enough for further comparisons, as the estimated nucleotide distance between *S. lignohabitans* and *S. novakii* was 0.55839

After the completion of the above-mentioned assembly, more *Sugiyamaella* raw genome sequences became available, including the ex-type strain of *S. novakii* NCAIM Y.00986 [71]. To compare our assembly with the new Illumina assembly, we performed a whole genome alignment with the Mauve aligner, in which we aligned the 238 unique Illumina contigs to our chromosome-level assembly. Thereafter, we reordered the Illumina contigs based on synteny relations using the Nanopore assembly as reference (Fig. 3A). The two assemblies were substantially similar, validating each other despite the differing sequencing technologies that they are based on (Fig. 3A; Table 2).

### Identification of potential mycotoxin eliminating enzymes in the newly assembled *S. novakii* genome

It was mentioned earlier that we have managed to find 30 enzymes for potential mycotoxin elimination in the proteome of *S. lignohabitans* and that *S. lignohabitans* is one of the closest relatives of *S. novakii*. As the whole genome of *S. novakii* was sequenced and assembled, we could perform further analyses to compare the two species and find candidate genes for mycotoxin eliminations in *S. novakii*, too.

To estimate the overall genome conservation between the two species, whole genome alignments were performed with Mauve and YASS (Fig. 3B). Although the Mauve alignment indicated that several rearrangement events could have occurred on the chromosomes of the species, the overall genome conservation seems to be high enough to justify the assumption of the similarity of the two species. Namely, the estimated nucleotide distance between *S. lignohabitans* and *S. novakii* was 0.55839, while the other three *Sugiyamaella* species scored 0.76487–0.870162 to *S. lignohabitans*. Besides, the estimation of genome conservation using YASS resulted in a 0.57492% genome similarity, which also strengthened our initial assumption regarding genome conservation (Supplementary Table S4).

Since the two species are substantially similar regarding their sequences and genome contents, the previously identified *S. lignohabitans* genes were used for similarity searches in the genome of *S. novakii*. Local tBLASTn searches in the raw genome sequence of *S. novakii* resulted in several hits (Supplementary Table S5). One putative trichothecene 3-O-acetyltransferase (AYT1) orthologous sequence for T-2, 10 aldo-keto reductase superfamily proteins for DON, 5 carboxylic ester hydrolases, and 2 ornithine aminotransferases for FB1 have been successfully identified (Supplementary Table S5). As for the ZEA mycotoxin, zearalenone hydrolase was not found either in the genome of *S. novakii*, but 7 carboxypeptidases, 4 peroxiredoxins, and 1 sterol 3-beta-glucosyltransferase enzyme could be found (Supplementary Table S5). The tBLASTn-identified sequences were overlapped with the found ORFs in the raw genome sequences of *S. novakii*.

Thus, the yeast strain *S. novakii* NCAIM Y.00986 encodes several different enzymes that may have roles in the degradation/modification of certain mycotoxins; however, further studies are needed to assess the true potential of those enzymes.

### Mycotoxin elimination studies

Given the demonstrated tolerance of *S. novakii* strains to *Fusarium* mycotoxins and the presence of numerous genes encoding potentially mycotoxin-degrading/modifying enzymes in the ex-type strain’s genome, we investigated the mycotoxin elimination capacity of these yeast strains. However, it is important to note that we have only performed tests for mycotoxin eliminations; the identification of modified or transformed mycotoxin products was out of the scope of this current study. That means if changes in the mycotoxin concentration were not detected with HPLC or ELISA assay after the yeast treatment, we considered no mycotoxin elimination to have happened.

**Fig. 4:**
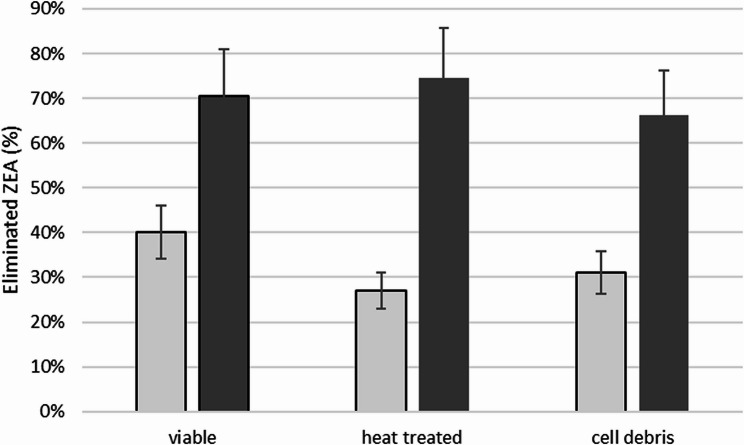
Zearalenone elimination capacity of *S. novakii* NCAIM Y.00986. Living cells, heat-inactivated cells, and cellular debris were tested during 1 and 6 h of incubation time at 25°C in PBS (2 ppm ZEA, *n* = 3). Increasing the incubation time substantially increased the toxin-eliminating capacity of the yeast. Unexpectedly, 6 h of incubation in PBS resulted in similar results of inactivated cells and cellular debris, as could be observed in the case of the living cells

Since the yeast strains exhibited tolerance against even 2 ppm mycotoxin concentrations, the elimination studies focused on that concentration. Elimination of DON and T-2 mycotoxins were not observed at all. The *S. novakii* NCAIM Y.00986 strain eliminated 26.08% ZEA and 15.89% FB1 from YPD broth during 1 h incubation at room temperature. Unexpectedly, in MXGB medium, neither ZEA nor FB1 eliminations could be detected. However, in PBS supplemented with 2 ppm mycotoxins, the NCAIM Y.00986 strain decreased only the amount of ZEA during 1 h (Fig. 4). Extending the incubation time from 1 to 6 h increased the ZEA elimination rate of the same cell preparations by approximately 30–40%. Accordingly, elimination of ZEA after 6 h of incubation could reach a spectacular 80% rate in PBS (Fig. 4). Since we have found several enzymes that may have potential in the elimination of the ZEA mycotoxin (Supplementary Table S5), we have performed further experiments to test this assumption. Accordingly, heat-inactivated cells and cell debris were also tested. Strikingly, 6 h of incubation in PBS resulted in similar outcomes as could be observed in the case of the living cells (Fig. 4). Consequently, the observed ZEA removal was more likely attributable to cell wall adsorption than to specific enzyme activity. Inexplicably, the other *S. novakii* strain (NCAIM Y.00987) was incapable of eliminating mycotoxins at all.

## Discussion

The current study focused on different yeast strains from the *Trichomonascaceae* family. Antimicrobial activity against *Fusarium* strains and mycotoxin tolerance and elimination capacity of those yeasts were tested.

Certain yeast species have the ability to release volatiles into the atmosphere or compounds into the substrate, which significantly inhibits the growth of different types of fungi [2, 5–7, 19–21]. Other yeasts can compete for the resources or for the limited space with the fungal species [5, 13–15]. One of the most obvious signs for microbial antagonism is the presence of a clear inhibition zone around the yeast colonies in which the tested fungal strains cannot grow [46]. Unexpectedly, none of the 29 examined yeast strains of the 10 different species proved to be good antagonists against *Fusarium* species in such terms. Although our tested yeast strains could not inhibit the growth of *Fusarium* species, they were able to produce colonies in the presence of the fungal species. Because none of the tested yeast strains exhibited strong antagonistic ability against *Fusarium*, microbial interactions were not tested further. Nevertheless, microbial antagonism and mycotoxin tolerance depend on different molecular mechanisms; the yeast strains may show good tolerance and elimination capacity for *Fusarium* mycotoxins, despite the failed inhibition ability.

However, the yeast strains showed substantially different sensitivity to DON, T-2, FB1, and ZEA mycotoxins. The strains of *Diddensiella caesifluorescens*, *Spencermartinsiella lignipurtidi*, *Blastobotrys indianaensis*, *Sugiyamaella paludigena*, and *Sugiyamaella chiloensis* were sensitive to the given mycotoxins, but certain strains of *Spencermartinsiella europaea* and *Sugiyamaella japonica* exhibited substantial tolerance to the tested mycotoxins. The most striking performances belonged to one strain of *Spencermartinsiella europaea* and the two strains of *S. novakii*, as they showed outstanding tolerance for most of the mycotoxins. These results also highlighted the fact that the mycotoxin tolerance was not necessarily a species-specific but rather a strain-specific trait.

It was also noticeable that *S. paludigena* and *S. chiloensis* were more sensitive to the presence of the mycotoxins than *S. japonica* and *S. novakii* were. We were curious to know whether the similarly behaving species were more closely related to each other or not. According to our phylogenetic analyses (considering the 26S rDNA of the yeasts), the mycotoxin-tolerant species are more closely related to each other than to the sensitive species. This minor observation may assist in identifying new candidates within the *Sugiyamaella* genus for additional mycotoxin testing; nevertheless, we do not propose a definitive correlation between phylogenetic distance and mycotoxin tolerance. We also noted that *S. lignohabitans* is one of the closest relatives of *S. novakii*. Although our phylogenetic analyses did not include all the described species of the *Sugiyamaella* genus and were based on only the LSU of the rDNA region, our results were in agreement with a more robust phylogenetic analysis [38].

The observed mycotoxin tolerance could indicate a possibility for mycotoxin degradation or neutralization of the toxic effect through the enzymatic or physical effect of the yeasts. Based on the processed literature, there are examples for all the tested *Fusarium* mycotoxins that different enzymes of certain microbes (bacteria, yeasts, or fungi) were successfully able to degrade, transform the mycotoxins, and reduce the amount of mycotoxins [2, 8, 10, 12, 24, 25, 28–33, 41, 65–70]. Since the two *S. novakii* strains were completely tolerant for *Fusarium* mycotoxins (except FB1), we supposed that the genome of this species may harbor a handful of enzyme-encoding genes participating in detoxification. Although yeasts are generally considered resistant organisms to many drugs and toxins via efficient efflux [72–74], in our case, the observed tolerance of the *S. novakii* strains was not the consequence of general resistance since most of the tested yeast strains proved to be sensitive to the different mycotoxins. Actually, *Fusarium* mycotoxins can be especially cytotoxic to yeast cells, too [12]. If a whole genome sequence for the species of interest is available, it is possible to search for genes encoding particular enzymes, which may have roles in the observed mycotoxin tolerance. For a preliminary analysis, we examined the gene pool of *S. lignohabitans*, which was not just one of the closest relatives of *S. novakii* but also had an annotated whole genome sequence available. We managed to identify 30 different enzymes in the proteome of *S. lignohabitans* that maybe in connection with mycotoxin tolerance and elimination. We have focused on mycotoxin-modifying and degrading enzymes only; general stress tolerance or detoxification genes were not considered. However, the identified 30 enzymes in the proteome of *S. lignohabitans* indicated that the genome of *S. novakii* might also contain several enzymes with mycotoxin-degrading potential.

Therefore, we have sequenced and assembled the genome of *S. novakii* NCAIM Y.00986, which also has 4 chromosomes similarly to *S. lignohabitans* [64]. Pairwise whole genome alignments further rationalized the comparisons of the two species. In the meantime, another whole genome sequence of the ex-type strain of *S. novakii* became available [71], which has validated our genome sequencing and assembly. Using the previously identified enzyme sets, tBLASTn searches were performed in the raw genome sequence of *S. novakii*, and we have found one or more putative orthologous coding genes for almost all the collected *S. lignohabitans* enzymes in the *S. novakii* genome. Consequently, *S. novakii* also has a decent enzyme-encoding gene set, which may contribute to the striking mycotoxin tolerance of the species. However, even the high sequence similarity of the enzymes cannot guarantee highly similar functions. For instance, the trichothecene 3-O-acetyltransferase TRI101 that is able to modify DON has different activity in *Fusarium graminearum* and in *F. sporotrichioides* [75].

Subsequently, we sought to determine if *S. novakii* could eliminate the selected mycotoxins. According to our results, significant elimination of DON, T-2, or FB1 was not observed; *S. novakii* could eliminate only the ZEA mycotoxin in a considerable amount. The structure of ZEA resembles that of estrogen, resulting in predominantly estrogenic effects that pose risks to animals and humans [76]. In yeasts, ZEA can cause chromosome and nucleus fragmentations, cell cycle arrest, disturb cell homeostasis, cellular metabolism, and fermentation [12, 77, 78]. Thus, a saprophyte yeast species, which is not just tolerant but also can eliminate the ZEA mycotoxin, may be a suitable candidate for the detoxification of feedstock. However, for such an application, it is imperative to understand the precise mechanism of action. There may be a chance that *S. novakii* can utilize ZEA as a nutrition source because in PBS the ZEA elimination efficiency was substantially better than in YPD or in MXGB broth. A further possibility is the enzymatic degradation of the mycotoxins, in which the degraded product was excreted. To find out if the observed elimination of ZEA was a consequence of targeted metabolic activity or not, heat-inactivated cells and cell debris were also tested as both terminate metabolic activity. Unexpectedly, both the inactivated cells and cellular debris could substantially reduce the amount of ZEA, similar to the active cells. Therefore, the observed decrease for ZEA may have been of adsorption type rather than targeted enzymatic degradation or metabolic utilization. It is known that certain materials (e.g., bentonites, diatomites, zeolites, or yeasts) are outstanding adsorbents for certain mycotoxins, whereas binding of ZEA is comparatively low [79, 80]. Thus, the adsorption type of ZEA reduction is a remarkable phenomenon on its own. The results of Yiannikouris and co-workers suggested that the β-d-glucan content of the yeast cell wall plays a crucial role in the ZEA adsorbing capacity [81]. This phenomenon could be a reason for the substantially different ZEA adsorbing ability of the two tested strains of *S. novakii*, since different strains of the same yeast species can have slightly different cell wall structures [81–83].

However, we should bear in mind that our investigations focused only on the elimination of the mycotoxins; the identification of modified or masked mycotoxin products was out of the scope of this current study. Given the remarkable tolerance of *S. novakii* to other mycotoxins (DON, T-2, and FB1) and the existence of various genes encoding potentially mycotoxin-modifying enzymes, future studies should focus on the understanding of the molecular basis of the striking mycotoxin tolerance of *S. novakii*. Moreover, to reveal the circumstances when those enzymes are transcriptionally active and assess particular enzyme activity and substrate specificity for additional mycotoxins or heterologous expression of the enzymes in model yeasts (e.g., *Saccharomyces cerevisiae*, *Pichia (Komagataella) pastoris*) could yield intriguing and beneficial results.

## Conclusions

The present study examined the microbial antagonistic potential, the mycotoxin tolerance, and the mycotoxin elimination capacity of several yeast strains belonging to the less-known *Trichomonascaceae* family. While the tested 29 yeast strains were proved to be inefficient for antagonistic purposes against different *Fusarium* species, some of them showed good mycotoxin tolerance against *Fusarium* mycotoxins. One of the best-performing yeast strains, the *Sugiyamaella novakii* NCAIM Y.00986, was not just highly tolerant to mycotoxins but was able to efficiently eliminate the ZEA mycotoxin from the substrate. The whole genome sequencing of *S. novakii* NCAIM Y.00986 revealed the presence of many genes potentially involved in mycotoxin elimination, but the strain eliminated ZEA more likely through cell wall adsorption rather than enzymatic degradation. This study highlights the potential of *S. novakii* for ZEA detoxification and emphasizes the role of yeast cell walls in mycotoxin mitigation strategies. According to that, future studies should focus on the exploration of potential applications in mycotoxin detoxification.

## Supplementary Information

Supplementary Material 1. The Excel Supplementary Tables file contains five sheets as follows: Table S1. Results of biocontrol tests of yeast strains against different *Fusarium* species. Table S2. Results of mycotoxin tolerance tests of the yeast strains. Table S3. Enzymes of *Sugiyamaella lignohabitans* that may have potential roles in mycotoxin tolerance and elimination. Table S4. Results of whole genome alignment created with YASS between *S. lignohabitans* and *S. novakii*. Table S5. tBLASTn results of *S. novakii* using certain enzyme sequences of *S. lignohabitans* as queries.

## Data Availability

Every piece of data generated or analyzed during this study is included in this published article (and its Supplementary Material). The genome assembly of *S. novakii* NCAIM Y.00986 was deposited to GenBank, accession number JBJGDZ000000000 (https://www.ncbi.nlm.nih.gov/nuccore/JBJGDZ000000000), annotation was deposited in FigShare (10.6084/m9.figshare.27640185). The raw sequencing reads are deposited under NCBI BioProject PRJNA1183396 (https://www.ncbi.nlm.nih.gov/bioproject/?term=PRJNA1183396).
